# Report of the Trial

**Published:** 1850-09

**Authors:** 


REPORT OF THE TRIAL. THE PEOPLE VS. DR. HORATIO N. LOOMIS, FOR LIBEL.

*Tried at the Erie Court of Oyer and Terminer, June,* 24, 1850.

In a bibliographical notice of Bennett on the Uterus, in the June No. of the Examiner, we incidentally alluded in terms of commendation to the recent effort of the Professor of Obstetrics in Buffalo Medical College to introduce the method of teaching midwifery by demonstration. It now appears that the information on which that opinion was based, was erroneous, as shown by the evidence brought forward at the late trial for libel, in "the People vs. Loomis;" we must therefore withdraw whatever countenance may have been given by the article mentioned, so far as regards *ocular* demonstration.

We must also protest against the necessity of exposing the female during the operation of turning, as advocated by one of the witnesses at the trial; indeed we cannot see under what circumstances it can be even admissible. It certainly is not the practice nor the doctrine taught where we were educated, that "exposure is a necessary element of the operation."

We have alluded to this pamphlet very briefly, merely to correct the opinion advanced by us under a wrong view of the case. We cannot but regret deeply that the matter was ever brought before the public at all; more especially as regards the statements made by one of the witnesses already referred to, in which we are sure he will find no sympathy from a very large majority of the profession.

